# All Impostors Aren’t Alike – Differentiating the Impostor Phenomenon

**DOI:** 10.3389/fpsyg.2017.01505

**Published:** 2017-09-07

**Authors:** Mona Leonhardt, Myriam N. Bechtoldt, Sonja Rohrmann

**Affiliations:** ^1^Department of Psychology, Goethe University Frankfurt Frankfurt, Germany; ^2^Department of Management and Economics, EBS Universität für Wirtschaft und Recht Oestrich-Winkel, Germany

**Keywords:** impostor phenomenon, *k*-means clustering analysis, Ward clustering, self-concept, self-presentation, strategic behavior, authenticity, strain

## Abstract

Following up on earlier investigations, the present paper analyzes construct validity of the impostor phenomenon. It examines the question whether the impostor phenomenon is a homogeneous construct or whether different types of persons with impostor self-concept can be distinguished on the basis of related characteristics. The study was conducted with professionals in leadership positions exhibiting a pronounced impostor self-concept (*n* = 183). Cluster-analytic procedures indicated the existence of two different types: one group which, in line with the literature (e.g., Clance, 1985), possessed traits classified as fairly unfavorable (“true impostors”) and another group which can be described as largely unencumbered (“strategic impostors”). The present study suggests two types of impostorism: “True” impostors characterized by the negative self-views associated with the construct definition, and more “strategic” impostors who seem to be less encumbered by self-doubt. It is assumed that “strategic impostors” are characterized by a form of deliberate self-presentation. Therefore, the impostor self-concept cannot principally be viewed as a dysfunctional personality style. This distinction should be more carefully considered in further research and in therapeutic interventions.

## Introduction

The term *impostor phenomenon* describes the intraindividual phenomenon that individuals experience themselves as inadequate and do not believe in their own abilities, even though they are objectively considered capable and competent due to their professional or academic accomplishments and qualifications (Clance and Imes, 1978). Moreover, they are convinced of having fooled their environment with respect to their capabilities and hence are afraid of being exposed as a fraud or impostor, once their environment has the opportunity to recognize their supposed incompetence (e.g., Harvey, 1981; Langford and Clance, 1993; Leary et al., 2000). Typically, they attribute success not to their own abilities, but to external factors such as hard work, timing, or luck (e.g., Harvey, 1981; Clance and O’Toole, 1987). They reject praise or recognition and belittle the importance of positive evaluations or of their achievements, since they view them as undeserved. As a consequence, factors such as appreciation, power and status, which are linked with success do not increase confidence in their own capabilities but, on the contrary, trigger fears of failure. These feelings differ from general self-presentation concerns. People experiencing the impostor phenomenon are not engaging in self-deception, believing they are better skilled than they displayed and they try to keep up displays of public perfection (e.g., Ferrari and Thompson, 2006). There is a strong and positive correlation between the impostor phenomenon and self-handicapping (e.g., Ross et al., 2001; Want and Kleitman, 2006), but individuals scoring high on impostorism more likely engage in self-handicapping to explain poor performance in retrospect rather than to reduce their efforts before task performance (Ferrari and Thompson, 2006). Contrary to initial assumptions of Clance and Imes (1978) this phenomenon does not seem to be gender-typical (e.g., Harvey, 1981; Cozzarelli and Major, 1990; Leary et al., 2000; Rohrmann et al., 2016). Up until now, substantial correlations have been observed between the impostor phenomenon and symptoms of depression (Kolligian and Sternberg, 1991; Chrisman et al., 1995), increased anxiety (Kolligian and Sternberg, 1991; Chrisman et al., 1995; Ross et al., 2001), lower self-esteem (e.g., Chrisman et al., 1995; Thompson et al., 1998), lesser expectations of success (Thompson et al., 1998), as well as unfavorable working styles such as perfectionism (Thompson et al., 2000) and task delegation behaviors of leaders (Bechtoldt, 2015). Moreover, recent studies revealed that the impostor phenomenon acts as an inner barrier to career development (Neureiter and Traut-Mattausch, 2016a) and is negative related to work-relevant outcomes, such as job satisfaction, or salary levels and promotions due to decreased career self-management factors (Neureiter and Traut-Mattausch, 2016b). Even though the impostor phenomenon is accompanied by various personality correlates, there is empirical evidence that the impostor phenomenon can be distinguished from related constructs such as anxiety, dysphoric mood or perfectionism and can therefore be considered as an independent construct (Rohrmann et al., 2016).

Originally, it was assumed that people experiencing the impostor phenomenon are afraid of being overestimated by their environment because they view themselves as less competent than others consider them to be (e.g., Clance and Imes, 1978). However, investigations by Leary et al. (2000) have shown that this fear does not result from a discrepancy between self- and other-evaluation, but that due to their overall negative self-evaluation persons with an impostor self-concept are generally concerned about possible negative evaluations of themselves or of their performance. Another line of research had shown that individuals also tend to present themselves in an unfavorable rather than a favorable light when they believe that negative self-presentation has a social value for them (e.g., Baumeister et al., 1979; Kowalski and Leary, 1990; Leary, 1995; cf. Leary et al., 2000). Leary and colleagues (ib.) thus assumed that the public self-incrimination of being incapable is both, a strategy and an essential facet of the impostor phenomenon. An investigation on this aspect showed that the impostor self-concept influenced expectations of affected persons concerning their own performance, their evaluation of the relevance of test results, as well as their appraisal of test validity, depending on whether their own prediction was to be made public afterward and on the performance the experimenter expected of them. When their predictions were public and the experimenter officially expected only a fairly bad performance, persons with high values on the impostor scale predicted their performance on the test to be significantly worse than individuals with low impostor values; they also belittled the significance of test results and expressed doubts concerning test validity. But, when they believed their predictions to remain anonymous, there were no differences between estimations of individuals with high and low values on the impostor scale: persons with an impostor self-concept expected themselves to show a similar performance as persons without impostor self-concept did and they downplayed neither the significance of the test nor its validity. Based on these findings the authors questioned the validity of the impostor self-concept. They hypothesized that different types might be differentiated within the group of persons with impostor self-concept: persons who really regard themselves as incompetent and show the corresponding behaviors and others whose negative self-presentation is primarily strategically motivated. The advantage of negative self-presentation lies in gaining likeability. This form of self-presentation signals caution and modesty, two socially valued traits (Blickle et al., 2012); and invalidating negative self-evaluations with a good performance will evoke more positive feedback than the opposite case.

So far, it has not been common to differentiate among persons with impostor self-concept. Although Harvey (1981) mentioned six types of so-called “impostors,” she does not refer to empirical investigations but formulates general assumptions on various characteristics. It is conspicuous that the different types (e.g., “the workaholic”) are characterized by an essential feature like perfectionism/over-doing, but are not described in relation to each other, so that there is no information on the features that differentiate them or that they share. Following up on the results of Leary et al. (2000), the present study therefore examined whether different types of persons with impostor self-concept could be distinguished. In line with earlier studies, we hypothesized that individuals reporting elevated levels of impostorism suffer from higher levels of anxiety and depression because of their fear of failure and their constant worries about being unmasked as a fraud (cf. Chrisman et al., 1995; Thompson et al., 1998; Ross et al., 2001). Attributing their previous successes to external conditions like luck, they negatively assess their own abilities to replicate these successes, resulting in low levels of self-efficacy. Their non-benevolent attribution style regarding their previous achievements associates with an external locus of control, and their pervasive negative self-views concur with low self-esteem (cf. Clance and Imes, 1978; Chrisman et al., 1995). These negative self-evaluations trigger a spiral of dysfunctional behaviors that ironically perpetuate the negative self-views: individuals with elevated levels of impostorism tend to report both, higher levels of perfectionism and procrastination at work (cf. Clance, 1985; Cowman and Ferrari, 2002). On the one hand, these seemingly incompatible habitual behaviors inoculate individuals with elevated levels of impostorism against the revision of their negative self-views because exerting themselves tirelessly in a perfectionist manner makes them attribute their successes to effort rather than ability. On the other hand, procrastinating tasks becomes more likely when they feel they cannot live up to their own perfectionist standards. Both behavioral tendencies, perfectionism and procrastination, are likely to associate with elevated levels of strain. Whereas these features have been mentioned as core indicators of impostorism, we hypothesized that these associations do not pertain to all individuals reporting elevated levels of impostorism. We hypothesized that downplaying one’s own abilities, for example, may serve the goal to favorably control one’s image rather than reflect truly negative self-convictions. First, it may signal modesty, which favorably associates with liking and even professional success (Blickle et al., 2012). Second, in case of failure, others will react more positively than they would if the individual had bragged about their abilities and not anticipated the likelihood of failure. The assumption that individuals may report elevated levels of impostorism for reasons of impression management is supported by earlier findings (Leary et al., 2000) suggesting that individuals with impostor self-concept evaluate themselves more positively in private than in public. If in fact some individuals strategically report elevated levels of impostorism, impostorism should be associated less strongly with negative self-convictions, dysfunctional task-related behaviors, and elevated levels of strain in this group. In sum, we investigated whether among individuals reporting elevated levels of impostorism, there are different subgroups to be differentiated. Based on previous research, we hypothesized that some individuals with elevated levels of impostorism evaluate themselves negatively, report dysfunctional task-related behaviors, and suffer from increased strain (*Hypothesis 1*). Besides, we hypothesized that there is a second group of individuals with elevated levels of impostorism whose self-views are significantly more positive, who report less dysfunctional task-related behaviors and lower strain (*Hypothesis 2*).

Originally, the impostor phenomenon was assumed to be a categorical construct (Clance, 1985) which does not differentiate between people diagnosed as impostors. As the present studies aimed to further inspect differences between people scoring high on impostorism, we treated impostorism as a dimensional trait (cf. Ferrari, 2005).

Whereas previous research predominantly assessed impostorism among student samples, we analyzed a sample of professionals in leadership positions. Given that these professionals were objectively successful as indicated by their career attainments, they formed more appropriate a group to study impostorism than student samples would.

## Materials and Methods

### Sample

In all, 242 persons (36.77% women) employed in leading positions (e.g., leadership experience, employee responsibility) in various economic sectors participated in the investigation. Potential participants were directly contacted via e-mail. For example, we were using contacts of institutions associated or cooperating with Frankfurt University (e.g., Scientific Society), the University’s alumni network und mailing lists of a network of women in leadership positions and a network of leaders in the financing and consulting sector. The cover letter included some information as well as a direct link to the survey. In addition, all contacted persons were asked to forward the request to acquaintances in leadership positions. As an incentive, the participants were offered to receive a comprehensive personality profile after evaluation of the data.

A minimum of one year employment in a leading position and the existence of at least one fellow employee were required to take part in the study. Participants (mean age: 44.30; *SD* = 9.02) were experienced executives who had held their position for an average of 10.73 years (*SD* = 8.24); the median number of fellow employees was eight. In all, *n* = 183 participants completed the entire questionnaire. All information was saved even when the survey was abandoned so as to also include data from incomplete questionnaires in the analysis. Therefore, internal consistencies reported below are based on sample sizes ranging from 183 to 242 participants. As the present study was conducted to analyze a sample of professionals in leadership positions, the question was whether the decision to drop out of the study was related to years of professional experience. In fact, participants who completed the questionnaire possessed longer leadership experience than those who dropped out (completed: *M* = 11.78; *SD* = 8.29; dropouts: *M* = 7.64; *SD* = 7.57; *t*(105.51) = 3.55 *p* = 0.001, 95% *CI*_Diff_[1.83, 6.45], *d* = 0.52). Congruent with this, participants who completed the entire questionnaire were older than those who abandoned it (completed: *M* = 45.39; *SD* = 8.49; dropouts: *M* = 40.78; *SD* = 9.82; *t*(88.43) = 3.23, *p* = 0.002, 95% *CI*_Diff_[1.78, 7.45], *d* = 0.50). There were no differences in gender or educational level.

### Procedure

Having received the link to the survey, the participants could answer the online questionnaire at any time using any computer with access to the internet. All participants received the online questionnaire in the same order. The instructions were standardized for the entire sample and thus warranted objectivity of the procedure. Completing the survey took 25 min on average.

### Instruments

If not stated differently, all responses were marked on six-point scales (1 = *does not apply at all*, 6 = *applies completely*), to keep the task as simple as possible and to produce differentiated responses by avoiding a middle category.

*Impostorism* was assessed with the 20-item Clance Impostor Phenomenon Scale (CIPS; Clance, 1985) in its German translation by Salm-Beckgerd (quoted in Clance, 1988; e.g., “When people praise me for something I’ve accomplished, I’m afraid I won’t be able to live up to their expectations of me in the future.”). CIPS was used to capture the impostor self-concept in a multifaceted way, assessing among other things the fear of being evaluated, the fear of failing to reproduce an achievement as well as the tendency to overestimate others (cf. Holmes et al., 1993; Chrisman et al., 1995). In addition, research has shown that CIPS reliably distinguishes persons with impostor self-concept from those without (Holmes et al., 1993). Participants were presented with a six-point scale (1 = *does not apply at all* to 6 = *applies completely*). The scale had an internal consistency of α = 0.91.

As indicators of positive self-evaluation, *core self-evaluations* were measured with the German translation of the Core Self-Evaluations Scale (CSES; Judge et al., 2003; Heilmann and Jonas, 2010). The CSES consists of 12 items (e.g., “I doubt my competence”) and measures the four traits self-esteem, self-efficacy, internal control conviction, and emotional stability. Internal consistency of the scale was α = 0.86.

Habitual *anxiety* and *depression* were assessed with the 20 items of the State-Trait Anxiety Depressions Inventory (STADI; Laux et al., 2013). They used a four-point scale (1 = *almost never*, 4 = *almost always*) to indicate how often the respective statement applied to them. Anxiety was measured with the two subscales agitation (five items, e.g., “I am easily tense”) and apprehension (five items, e.g., “I worry about problems that might occur”). For both scales internal consistency was α = 0.82. Depression was assessed with the two scales dysthymia (five items; e.g., “I feel empty”) and its bipolar opposite, euthymia (five items; e.g., “I enjoy life”). Internal consistencies were α = 0.80 (dysthymia) and α = 0.90 (euthymia).

*Perfectionism* and *procrastination* were measured to capture participants’ habitual task-related behaviors. To measure perfectionism we used the Frost Multidimensional Perfectionism Scale (Frost et al., 1990) in the German translation by Stöber (1995a, unpublished; “I expect higher performance in my daily tasks than most people.”). For economic reasons the 35 items of the original FMPS-D were reduced to 17. Criteria for selecting items were the psychometric parameters reported in the literature as well as aspects of content. We chose the subscales “Personal Standards” and “Concern over Mistakes and Doubts” (Stöber, 1995b, unpublished); the latter was shortened to 10 items (FMPS: 9, 14, 17, 18, 21, 23, 25, 28, 32, 34). Internal consistency of the scale was α = 0.85. To assess procrastination we used the nine items with the highest item-part whole correlations from the Tuckman Procrastination Scale (Tuckman, 1991) in the German translation by Stöber (included items: 1, 3, 4, 11, 12, 13, 14, 15, 16). To assess procrastination we used the Tuckman Procrastination Scale (TPS, Tuckman, 1991) in the German translation by Stöber (e.g., “When I have a deadline, I wait till the last minute.”). The 16 items of the original TPS-D were reduced to the nine items with the highest item-part whole correlations (included items: 1, 3, 4, 11, 12, 13, 14, 15, 16). Internal consistency of this scale was α = 0.92.

The Irritation scale (IS; Mohr et al., 2007) was used to assess the extent of *strain* (stress). The construct to be measured, irritation, describes a psychological exhaustion that results directly from one’s work and is too pronounced to be relieved with ordinary breaks (Mohr and Rigotti, 2001, e.g., “After work it is difficult for me to unwind”). The scale comprised eight items and had an internal consistency of α = 0.88.

Apart from these instruments we assessed demographic variables (age, gender, type of employment, duration of leadership experience, and number of fellow employees).

## Results^1^

**FIGURE 1:**
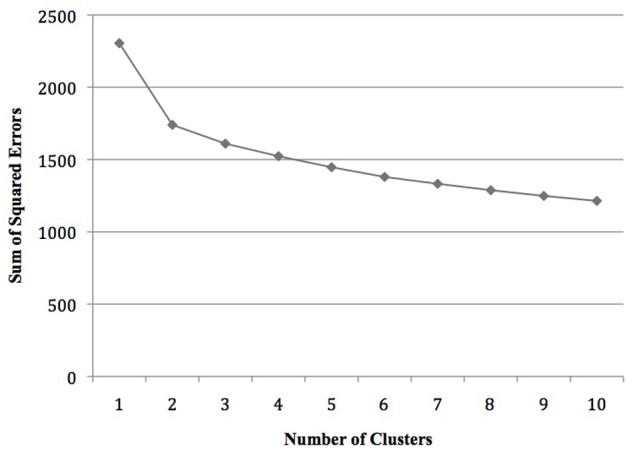
Heterogeneity measures across the final 10 fusion steps (*n* = 178).

To examine whether different groups, i.e., different types of persons with impostor self-concept, could be distinguished and, if so, which traits characterized them, an agglomerative hierarchical cluster analysis was conducted across the entire sample (*n* = 183). Prior to the analysis five outliers were eliminated through single linkage clustering, and variables were transformed into z-scores. A cluster analysis was then conducted with the help of the Ward procedure; this yielded a two-cluster solution (cf. **Figure 1**).

**FIGURE 2:**
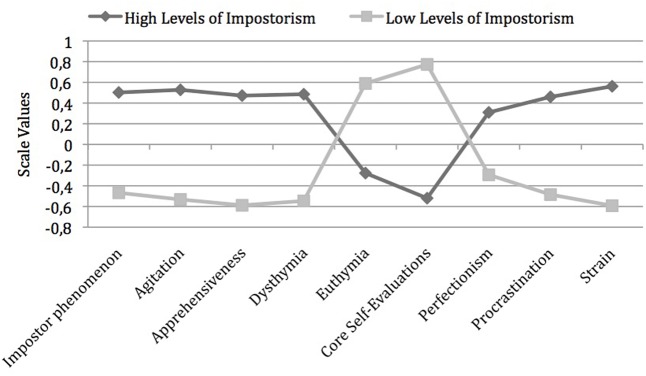
Cluster solution based on Ward procedure after optimization with *k*-means algorithm (*n* = 178).

To test the cluster solution obtained, cluster centroids from the Ward solution were used as starting values for the *k-*means algorithm (cf. Funke, 1990 quoted in Moosbrugger and Frank, 1992) and the number of clusters was defined as *k* = 2. The final cluster solution was reached after four iterations. After the *k-*means procedure 96 persons were in the first cluster and 82 persons in the second one. In all, there was high agreement in allocations (85.39%), so that the two-cluster solution was retained (cf. Funke, 1990). The trait levels in the two clusters are shown in **Figure 2**.

These results indicate a clear differentiation of two clusters. The mean cluster centroids of impostorism were *M* = -0.47 versus *M* = 0.50 respectively. Thus, there was one cluster with persons scoring high on the impostor scale (“impostor self-concept”; Cluster 1) and another with persons scoring low (“non-impostor self-concept”; Cluster 2). As mentioned above, more than half of the participants (53.93%) were in the first cluster. It is noteworthy that the profile lines of the two clusters form a mirror image; accordingly the difference in all trait levels between the two clusters was highly significant as to be seen from **Table 1**, the differences between groups were calculated with *t*-tests for independent samples; α-inflation was controlled by Bonferroni correction.

**Table 1:** Differences in trait levels between the clusters “impostor self-concept” (Cluster 1) and “non-impostor self-concept” (Cluster 2).

	*M*_*Group*1_	*SD*	*M*_*Group*2_	*SD*	*t*	*df*	95% *CI_Diff_* (*M*_*Group*1_–*M*_*Group*2_)	*d*
Agitation^1^	0.53	0.88	-0.53	0.65	9.24^∗^	172.82	0.83 to 1.29	1.37
Apprehensiveness^1^	0.47	0.82	-0.59	0.57	10.14^∗^	168.75	0.85 to 1.27	1.50
Dysthymia^2^	0.49	0.91	-0.55	0.64	8.79^∗^	170.03	0.80 to 1.26	1.32
Euthymia^2^	-0.28	0.78	0.59	0.64	-8.19^∗^	175.77	-1.08 to -0.66	-1.22
Core self-evaluations	-0.52	0.67	0.77	0.62	-13.31^∗^	174.79	-1.49 to -1.10	-1.99
Perfectionism	0.31	0.91	-0.29	0.85	4.58^∗^	174.62	0.34 to 0.86	0.68
Procrastination	0.46	0.92	-0.48	0.79	7.39^∗^	175.99	0.69 to 1.19	1.09
Strain	0.56	0.82	-0.59	0.69	10.11^∗^	175.99	0.93 to 1.38	1.52


**n* = 178; ^1^subcomponents of anxiety; ^2^subcomponents of depression; 95% *CI_*Diff*_* = 95% confidence interval of difference between cluster means. ^∗^*p* ≤ 0.001.*

With respect to the demographic variables a significant difference emerged only for sector of employment: persons scoring high on the impostor scale (Cluster 1) were employed with significantly higher frequency in civil service, while the others were more frequently employed in the private sector (*t*(167.64) = 2.54 *p* < 0.012, 95% *CI_Diff_*[0.03–0.24], *d* = 0.45); applying the Bonferroni method to control for α-inflation, however, the difference was not significant. As for trait levels, the following picture emerged for the two groups: persons without impostor self-concept (Cluster 2) matched the picture of mentally healthy executives; i.e., they exhibited the different traits to degrees that can be regarded as favorable. They did not report elevated levels of impostorism, in other words, did not show pronounced self-doubts or fear of failure, and were neither anxious nor depressive. They often experienced positive emotions and described themselves as emotionally stable. In addition they reported generally positive core self-evaluations. Trait levels of their working styles can also be considered positive: they described themselves as not prone to postponing pending assignments and tasks. Moreover, they described themselves as non-perfectionist. Thus, they did not set themselves overly high standards and did not feel the need always to come out best in all different areas. Consequently, they did not feel stressed or particularly strained and burdened by their work.

According to the profile patterns, the opposite picture emerged for persons scoring high on the impostor scale (Cluster 1); this picture largely matched the characteristics of persons with impostor self-concept described in the literature (e.g., Clance, 1985): individuals scoring high exhibited significantly more anxiety than those from the other cluster. In addition, they tended to have dysphoric moods and negative emotions. Accordingly they experienced positive emotions only infrequently and reported a generally negative self-evaluation. Participants of this type indicated that, under stress, they easily lose their balance and that they were overall more sensitive than others. They also had perfectionist standards for themselves coupled with high degrees of commitment. At the same time they tended to postpone tasks. High degrees of experienced stress and strain marked this working behavior as overall unfavorable; persons with pronounced impostor self-concept described themselves as considerably more stressed than individuals without such feelings.

To test whether the two-cluster solution obtained could be validated, the sample was randomly divided in halves. First, a cluster analysis was calculated for one subsample (*n* = 94). As before, to determine the number of clusters and the corresponding cluster centers the Ward procedure was applied. Again, this yielded a two-cluster solution. We employed a *k-*means procedure to check and, if necessary, optimize the cluster solution obtained. The final cluster solution was reached after two iterations. After the *k-*means procedure 55 persons were in Cluster 1 and 39 in Cluster 2. As in the whole sample, one cluster with persons scoring high on the impostor scale (“impostor self-concept”; Cluster 1) was distinguished from another cluster with persons scoring low (“non-impostor self-concept”; Cluster 2). Regarding trait levels within clusters, the results were similar to those based on the whole sample.

Another cluster analysis was calculated for the second subsample (*n* = 84), with the number of clusters defined as *k* = 2, but without specifying cluster centers as initial values. We checked whether the cluster solution found in the first subsample could be replicated by calculating correlations between the cluster solutions of the two samples. The cluster centers of all trait levels in the total of four clusters formed the basis for calculating correlations. The results are shown in **Table 2**.

**Table 2:** Correlation matrix between the two-cluster solutions.

		Second subsample	
		**1**	**2**
**First subsample**	1 (“Impostor self-concept”)	0.955^∗^	-0.943^∗^
	2 (“Non-impostor self-concept”)	-0.981^∗^	0.948^∗^


*^∗^*p* < 0.01.*

The correlation matrix indicates nearly perfect positive correlation between the convergent clusters and nearly perfect negative correlation between the discriminant clusters. Thus, the results lend support both to the two-cluster solution obtained in the first random subsample and the results obtained in the whole sample.

**FIGURE 3:**
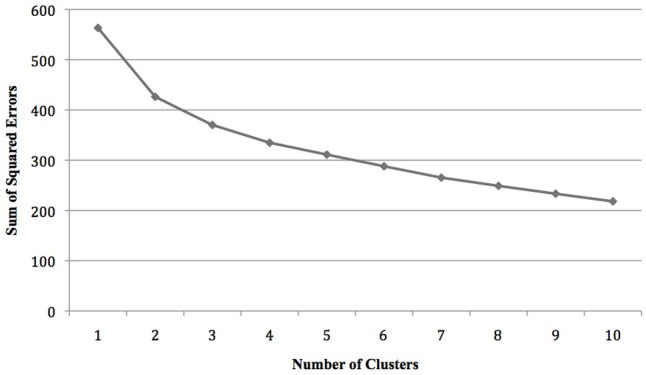
Heterogeneity measures across the final 10 fusion steps (*n* = 48).

To check whether persons experiencing symptoms of impostorism could be further differentiated into different types, another cluster analysis was conducted which paralleled the prior one. This analysis included only those participants whose raw impostor sum scores were above the sample median (CIPS scale score ≥ 49.8^2^). This sample (*n* = 89) was randomly divided into two subsamples, in order to validate in a second step the cluster solution obtained in the analysis of the first subsample with the help of the second one. To determine the number of clusters and the corresponding cluster centers the Ward procedure was applied to the first subsample (*n* = 48). Again, this yielded a two-cluster solution (cf. **Figure 3**).

**FIGURE 4:**
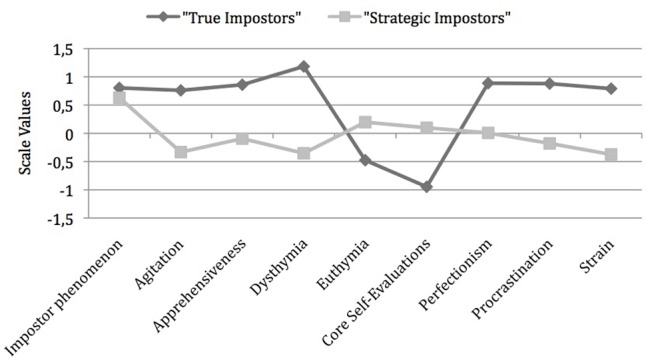
Cluster solution based on Ward procedure after optimization with *k*-means algorithm (*n* = 48).

As before, we employed a *k-*means procedure to check and, if necessary, optimize the cluster solution obtained. The final cluster solution was reached after three iterations. After the *k-*means procedure 22 persons were in Cluster 1 and 26 in Cluster 2. In all, agreement in allocations was very high (95.83%). Variable scores of the two clusters are shown in **Figure 4**.

Mean levels of impostorism (*z*-scores) were 0.80 (*SD* = 0.42) in Cluster 1 and 0.63 (*SD* = 0.43) in Cluster 2. This difference was small and non-significant, *t*(45.21) = 1.42, *p* = 0.16, 95% *CI_Diff_*[0.07–0.42], *d* = 0.40). For example, raw sum scores ranged from 50 to 74 in Cluster 1 and from 53 to 76 in Cluster 2. Accordingly, the level of impostorism was comparable in both clusters, which one might expect to concur with similar profiles. Instead, there were, however, large differences between both groups: participants in Cluster 1 experienced clearly negative drawbacks and overall exhibited rather unfavorable trait levels. These persons closely resembled general descriptions of individuals with impostor self-concept in the literature. Therefore, *Hypothesis 1* was supported. Taking up the term used by Leary et al. (2000) this group will be referred to as “true impostors.” Except for similarly high levels of impostorism the second group exhibited trait levels that can generally be regarded as favorable or inconspicuous. Persons allocated to this cluster did not show the expectable trait levels of persons with impostor self-concept, but rather resembled individuals without impostor self-concept. These findings supported *Hypothesis 2*. Again following Leary et al. (2000), this group will be referred to as “strategic impostors.” Individuals of this type, who report an impostor self-concept to a similar extent as “true impostors” but otherwise lack the traits associated with it, have not yet been described in the context of the impostor phenomenon. The two groups showed no significant differences in their demographic variables. Differences in trait levels between the two types of impostors will be described in detail below. As to be seen from **Table 3**, the differences between groups were calculated with *t*-tests for independent samples; α-inflation was controlled by Bonferroni correction.

**Table 3:** Differences in trait levels between impostor subgroups.

	*M*_*Group*1_	*SD*	*M*_*Group*2_	*SD*	*t*	*df*	95% *CI_Diff_* (*M*_*Group*1_–*M*_*Group*2_)	*d*
Agitation^1^	0.76	0.80	-0.33	0.68	5.05^∗∗^	41.21	0.66 to 1.53	1.47
Apprehensiveness^1^	0.86	0.79	-0.09	0.71	4.33^∗∗^	42.58	0.51 to 1.39	1.27
Dysthymia^2^	1.18	0.79	-0.35	0.67	7.15^∗∗^	41.22	1.10 to 1.97	2.09
Euthymia^2^	-0.48	0.78	0.19	0.75	-3.02^∗^	44.32	-1.12 to -0.22	-0.88
Core self-evaluations	-0.95	0.52	0.09	0.70	-5.90^∗∗^	45.39	-1.40 to -0.69	-1.69
Perfectionism	0.89	0.83	0.01	0.81	3.69^∗∗^	44.38	0.39 to 1.36	1.07
Procrastination	0.88	0.91	-0.18	0.88	4.07^∗∗^	44.19	0.53 to 1.58	1.18
Strain	0.79	0.65	-0.37	0.61	6.38^∗^	43.46	0.79 to 1.53	1.84


**n* = 48; ^1^subcomponents of anxiety; ^2^subcomponents of depression; 95% *CI_*Diff*_* = 95% confidence interval of difference between cluster means. ^∗^*p* ≤ 0.01; ^∗∗^*p* ≤ 0.001.*

### Group 1 (“True” Impostors)

As mentioned above, this type largely matches the general description of persons experiencing the impostor phenomenon. Individuals allocated to this cluster showed high levels of anxiety; they experienced largely negative and few positive emotions. Persons from this group reported highly negative self-evaluation. In their habitual task-related behaviors they tended toward high degrees of perfectionism as well as toward postponing pending tasks. The work-related stress and strain they described can be regarded as very high.

### Group 2 (“Strategic” Impostors)

Even though the two groups did not differ significantly in the feature “impostor self-concept,” i.e., members of both groups reported experiencing the impostor phenomenon to similar degrees, persons of the second type differed markedly from “true impostors.” Different from the first group and contrary to expectations concerning typical trait levels associated with the impostor self-concept, persons of the second type described themselves as not particularly anxious and not prone to dysphoric moods. They reported experiencing positive emotions and showed a tendency toward positive self-evaluation. With respect to their habitual task-related behaviors, as opposed to Cluster 1, persons of the second type stated that they did not set themselves particularly high standards for their performance. The same was true for the tendency to postpone pending tasks: individuals in this group showed no tendency toward procrastinating behavior. In all they described themselves as feeling neither particularly stressed nor strained by their work, in other words as not being under stress. With respect to all these features they differed significantly from individuals of the first type (*p* < 0.01). As to be seen from **Table 3**, effect sizes for all variable differences were large except for euthymia (Cohen, 1988).

To test whether the two-cluster solution obtained could be validated, as before, another cluster analysis was calculated for the validation sample (*n* = 41), with the number of clusters defined as *k* = 2, but without specifying cluster centers as initial values. After the *k-*means procedure 21 persons were in Cluster 1 and 20 in Cluster 2. Again, we checked whether the cluster solution found in the first sample could be replicated by calculating correlations between the cluster centers of all trait levels in the total of four clusters of the two samples. It was assumed that the cluster centers of the clusters in the analysis sample would correlate highly with their corresponding cluster centers in the validation sample but correspondingly little with the cluster centers of the other clusters. The results are shown in **Table 4**.

**Table 4:** Correlation matrix between the two-cluster solutions.

		Validation sample	
		**1**	**2**
**Analysis sample**	1 (“True impostors”)	0.93^∗^	-0.29
	2 (“Strategic impostors”)	-0.02	0.69^∗^


*^∗^*p* < 0.01.*

The correlation matrix indicates that there was a nearly perfect positive correlation between Cluster 1 of the analysis sample, the “true impostors,” and the first cluster of the validation sample. Moreover, there was a medium negative correlation with the cluster center of the second cluster (“strategic impostors”) from the validation sample. Accordingly, the second cluster of the analysis sample exhibited only a very weak negative correlation with the first cluster (“true impostors”) of the validation sample, whereas correlation with the second cluster in this solution was high. Thus, one cluster each of a given cluster solution showed a high positive correlation with only one cluster of the other sample. In all, the results document that the two-cluster solution obtained in the first random sample could be replicated.

## Discussion

The feelings and fears of persons experiencing the impostor phenomenon appear extremely paradox in view of their behaviors: on the one hand, these individuals describe their greatest fear as being exposed as incompetent, less intelligent and thus as a phony; on the other hand they belittle their achievements, reject praise and appreciation of their performance and even tend to deny evidence or facts that contradict their assumed incompetence and ultimately accuse themselves of imposture (cf. Clance, 1985). This behavior does not create the impression that the impostor self-concept is based on fears of being exposed and unmasked (cf. Leary et al., 2000). To inspect these inconsistencies, the present study investigated whether different types of persons experiencing symptoms of impostorism could be identified. In a sample of managers, two groups could be discerned: “true” impostors reporting high strain and suffering from pervasively negative self-views versus more “strategic impostors” largely unaffected by psychological impairments. These “true impostors” apparently form the group originally described by Clance and Imes (1978), i.e., persons who really doubt their own competence, attribute success externally and believe that they deceive others regarding their achievements. Accordingly, they suffer from the corresponding impostor feelings. On the other hand, there are individuals who claim to experience the impostor phenomenon even though they do not have the corresponding self-perception. This is then a strategic form of self-presentation employed to appear more modest and to keep others’ expectations concerning one’s abilities as low as possible. These results are in line with previous findings by Leary et al. (2000) which questioned the conception of the impostor phenomenon. However, whereas Leary and colleagues analyzed students, this study analyzed professionals with managerial responsibilities. Thus, their career attainments met a core requirement to study impostorism (cf. Clance and Imes, 1978).

In the sample as a whole, executives with impostor self-concept could be distinguished from those without a corresponding self-perception. Individuals without impostor self-concept showed trait levels that matched the image of psychologically healthy leaders with efficient working styles, while persons with pronounced impostor self-concept overall exhibited unfavorable trait levels, in line with descriptions in earlier investigations. These results are notable in that they identify two completely different groups, yet they do not allow for statements about different types of persons with impostor self-concept. Therefore another cluster analysis was conducted, including only those executives with high values on the impostor self-concept scale. In line with the assumptions of Leary et al. (2000) we identified a group exhibiting the expected unfavorable trait levels and another group where the impostor phenomenon was not accompanied by the typical pattern of traits. The latter persons appeared overall carefree and unstressed. This finding raises the question why some individuals report impostor feelings when they are obviously not affected by the unfavorable traits usually associated with the impostor phenomenon. They have a fairly positive self-evaluation, are not particularly anxious or; they show neither especially perfectionist nor procrastinating working styles and do not experience increased stress or strain by their work. We assume that these “strategic impostors” report impostor feelings to profit from an advantage of attribution. They downplay their achievements and abilities in order to keep others’ expectations low and to turn out successful despite their assumed incompetence. Different from “true impostors” they do not internalize their behavior but are aware of their competences. In them, the impostor phenomenon is a strategic form of self-presentation rather than actual self-perception (cf. Leary et al., 2000). This conclusion is supported by earlier research which documents that persons present themselves in an unfavorable rather than a favorable light when they believe that a negative self-presentation has a social value for them (e.g., Baumeister et al., 1979; Kowalski and Leary, 1990; Leary, 1995; cf. Leary et al., 2000). Future research, however, is warranted to gain further and more direct insights into what motivates this group of impostors to present themselves in an unfavorable light. At the same time, there is a group of individuals among people scoring high on impostorism haunted by profoundly negative self-views. These individuals may seek counseling and may have contributed to the emergence of the impostor phenomenon in the academic literature. Coaching- and psychotherapeutic techniques should aim to change their dysfunctional thoughts and self evaluations as well as dysfunctional kinds of working styles (e.g., perfectionism or procrastination). These interventions are meant to reduce psychological strain and to improve life quality of the subjects suffering from the imposter phenomenon.

### Limitations

While participants denoted as “true” and “strategic” impostors did not differ significantly with regard to their level of impostorism, so-called “true” impostors scored slightly higher. One may argue that this small difference accounted for the differences between the two clusters’ profiles. However, the differences were large – in fact, 6 of 8 (75%) effect sizes *d* were greater than 1. Arguing that these differences were due to the small differences in impostorism would suggest something like an “all or nothing” mechanism: if participants’ scores exceed a critical threshold, their psychological profile would change tremendously. Whether the dimensional view of impostorism dominating in the literature should be abandoned in favor of a typology of impostorism, as suggested decades ago (see Harvey, 1981), warrants further clarification. Also, future research with larger samples of professionals is desirable to further investigate the typology suggested here. Nonetheless, several features support the validity of our findings: first, the clusters differentiating between individuals with higher levels of impostorism were clearly separate, as they did not correlate or correlated negatively respectively. This feature lends support to the validity of our results, as the stability of clusters is dependent on the degree of overlap between clusters. If clusters are clearly separate, even sample sizes of 50 participants may yield stable results (Bacher et al., 2010, p. 303). Second, the findings were congruent with previous assumptions by Leary et al. (2000) who suggested that strategic impression management is a component inherent to impostorism. However, their analyses derived from student samples whereas we analyzed professionals who were successful in their jobs as defined by objective criteria like managerial responsibility. As professional success is a defining characteristic of impostorism, this feature lends additional support to the external validity of our results.

To sum up, persons with impostor self-concept form a heterogeneous group and the construct needs to be considered in a more differentiated way in future research. The results reported here suggest that, in about one half of the persons concerned, the impostor phenomenon constituted a form of self-presentation rather than actual self-perception. Therefore, the impostor self-concept cannot principally be viewed as a dysfunctional personality style related to strain and pervasively negative self-views, for some impostors lack the typical disadvantages associated with the self-concept. This distinction should be more carefully considered in further research and in the investigation of distinct professional and health-related consequences of the different types of impostorism.

## Ethics Statement

This study was carried out in accordance with the recommendations of standard ethical principles (privacy and protection of individual data, participant recruitment and briefing, participant distress), Ethics Committee of the Department of Psychology and Sports, Goethe University Frankfurt with written informed consent from all subjects. All subjects gave written informed consent in accordance with the Declaration of Helsinki. The protocol was approved by the Ethics Committee of the Department of Psychology and Sports, Goethe University Frankfurt.

## Author Contributions

ML substantially contributed to the conception and design of the work, interpreted the data, wrote the manuscript, and revised it critically for important intellectual content. MB supervised the design of the study and data collection and contributed to data analysis as well as writing and revising the manuscript. SR contributed to designing and conducting the study, analyzing the data as well as writing the manuscript. All authors contributed to and approved this final version of the manuscript.

## Conflict of Interest Statement

The authors declare that the research was conducted in the absence of any commercial or financial relationships that could be construed as a potential conflict of interest.

## Footnotes

1 The dataset can be openly accessed on the Open Science Framework under the heading of the present paper (https://osf.io/xrje7/).

2 This score exceeded the cut-off score suggested by Clance (1988) to differentiate between those unaffected by impostorism from those who experience symptoms of impostorism. To compare our sample with these cut-off scores we transformed Clance’s (1988) cut-off scores based on five-point response scales to the six-point scale scores used in this study. However, we just loosely refer to these cut-off scores because they were not derived from German samples.
